# The Middle Term

**DOI:** 10.1590/S1677-5538.IBJU.2017.04.01

**Published:** 2017

**Authors:** Stênio de Cássio Zequi

**Affiliations:** Editor Associado, International Braz J Urol. Divisão de Urologia do A.C. Camargo Cancer Center. Fundação A. Prudente, São Paulo, Brasil

As it is been daily debated, the dilemmas on to treat or not to treat patients with localized prostate cancer are under the scope of the “Difference of opinion section”: Between two extremes: the radical whole gland treatments versus the active surveillance protocols, the “middle term”, probably will be arise from judicious and individualized patient selection. Using biomolecular markers, nomograms, modern magnetic resonance evaluations and the individual patient preferences and payment capacities, the urologists will be able to decide a personalized approach’s, varying from traditional whole gland therapies, passing by the emerging focal treatments until exclusive surveillance. Concomitantly, in the future, less patients will be overdiagnosed and overtreated. Our readers will be able the get their self “middle term decisions”, after reading the favorable arguments (Dell´Oglio and Sanchez Salas, from Paris) and contrary to treatment (Schulman and Polascyck, from North Carolina).

Another area of intense discussion regarding prostate cancer is the treatment of the primary tumors in the metastatic scenario, which was addressed in an extensive meta-analysis by Carneiro et al. (page 588).

A Turkish study showed that patients with non-muscle invasive bladder cancer are not correctly warrant about the importance of the smoking cessation and the risks of the disease progression. Authors reinforced that urologists, which are the closest help professionals in contact with these patients, can advice them and their relatives regarding the risks of tobacco exposure on urinary bladder neoplasms and the access to smoking cessation programs (page 607).

Karguzel et al. report a new renal cell carcinoma plasmatic biomarker, the SCUBE-1, that was compared to IX Carbonic Anidhrase and urokinase plasminogen activator receptor but in a limited case-control series (< 50 individuals), deserving future internal and external validations (page 638).

Moving for renal transplantation, the group from Bangalore, India, analyzed in 243 laparoscopic donor nephrectomies, their experience with patients presenting unusual venous anatomies (retro-aortic and/or surrounding aortic renal veins). Using angio CT in the surgical planning, the laparoscopic approach was secure.

Between the modern endourological approaches and the extracorporeal shockwave lithotripsy, the percutaneous nephrolithotomy (PCL) is well stablished and seems a “middle term” treatment that has maintained its role in the urinary stone management. Sometimes, the patient’s body habitus, body masses index, and the extremes of age, can influence the PCL results as in prone or supine position. These questions were evaluated in three international studies in this issue of Int Braz J Urol (pages 679, 698 and 704).

Greek researchers demonstrated that women with overactive bladder treated with long action teltoredine scored higher levels of sexual function (measured by the Female Sexual Function Index (FSFI)), after the medication in comparison with before the drug intake.

Ankylosing Spondylitis (AS) is a male prevalent rheumatic affection, that negatively impact the wellness and quality of life of much men. Interestingly, Santana et al., from Curitiba, Brazil detected through the use of diverse specific questionnaires, a high prevalence of erectile dysfunction in AS population. These data suggest to the urologists a proactive posture regarding erectile function during the anamneses of these rheumatic men.

Stênio de Cássio Zequi, MD, PhD Editor Associado, International Braz J Urol Divisão de Urologia do A.C. Camargo Cancer Center Fundação A. Prudente, São Paulo, Brasil

